# Physiological Antagonism the Therapeutic Law of Cure

**Published:** 1879-08

**Authors:** Electus B. Ward


### Selections.

### PHYSIOLOGICAL ANTAGONISM THE THERAPEUTIC LAW OF CURE.

By ELECTUS B. WARD, M.D.

The natural instinct of the human mind is to reduce questions of science to definite laws. In medical science this has led to endless speculations and to most fanciful theories. Narrow-minded theorists reproach rational medical science with having no definite and universal law of cure. We are charged with being unscientific, irrational, and having no guiding principle to govern us in the treatment of disease. To those who have given the subject but little study this object carries with it seeming weight. Men and women naturally take to a

"system" that is said to be definite, universal, and exact in its application, however, absurd from the nature of the case such pretentions may be. We naturally incline in medicine, as in theology, to adopt creeds. They furnish a kind of mental and moral support to those that are content to follow, not to lead.

Medical science in the earlier stages of its history, was largely made of such speculative creeds and theories; and now there is a kind of non-rest in the popular professional mind for a creed, or for some universal well ascertained law of cure. This leads us to ask, is it possible, in the present state of medical science, to make any approach to such general law on a scientific basis, and include, at the same time, the fragments of all creeds and systems that have in their elements of truth? Can we, in other words, agree upon any formulated statement that is sufficiently broad and comprehensive to include everything that curatively affects the human organism, while, at the same time, it commits us to none of the one-idead speculative theories of the schools? And above all, can we formulate a law of cure that tends at all times to a sound and rational practice? To this possibility I make, with some degree of hesitation, my brief communication.

That curative remedies sometimes act anti-pathically, sometimes allo-pathically and sometimes homœo-pathically, there cannot be reasonable doubt; whether by altering the chemical constitution of the fluids, or modifying the sensibilities of the solids, we observe the operation of the same general law, namely, that of *physiological antagonism to the morbid* condition of which disease is but a manifestation. This is the central thought of my paper, and the facts and reasonings presented, will be good or bad, speculative or otherwise, as they may tend to strengthen or weaken the statement made in my text.

It is hopeful, I think, for the future of pathology that we fall back more and more in our explanation of disease on principles drawn from physiology; morbid action is only modified vital action—vital action acting under the disturbed condition of life; and I regard it equally hopeful for therapeutics that the action of remedies may be explained on the same general principle. For it is clear to my mind that drug action and diseased action from other causes may be studied from the same general standpoint. Drug action becomes diseased action when it does not act in harmony with nature.

By the title of my paper, it will be seen that I start with the proposition, believed to be true, that there is a physiological antagonism naturally and essentially existing between remedies which act curatively and the abnormal processes which take place in the human body; otherwise, disease, unless cured by unaided nature, will continue until the organism is destroyed. A condition in the system different from the disease, whether that be similar action, substituted action, or opposite action, must take place in order to cure, and this condition or action, whatever it may be or however it may act, I have ventured to call a condition of antagonism—antagonism to the morbid element which disturbs the organism; it must be so from the very nature of the action itself.

But this different condition is not produced by drugs alone, nor by general constitutional states. The action of one special disease on another furnishes many striking illustrations of the general law of antagonism. Thus a new disease we know sometimes cures an old one. Neuralgia of the fifth pair is often cured by erysipelas; glycosuria disappears during a course of typhoid fever; carbuncle has been known to stop diabetes; insane persons are said to loose their insanity during an attack of Asiatic cholera; small pox will, for the time, remove inveterate psoriasis; chronic diseases as a class, if in activity, antagonize continued fever or cholera; Esquiroll states that epileptics are attacked in small proportion and with slight severity by typhus; Louis has shown that typhoid fever rarely or never originates in the course of any acute affection; it is proven that ague prevents or greatly modifies the development of continued fever and of phthisis; certain heart diseases also antagonize tuberculous disease—notably mitral stenosis of left heart; gout and acute articular rheumatism are said to be incompatible. Ambrose Blocklock, surgeon major of the Madras army, believes also that "persons who had well marked cow-pox at no distant period are fully protected from Asiatic cholera;" he fixes upon five years as the limit of its action, and says after twenty years' residence in India he has seen no exception to the statement made.

A past disease has also been frequently observed to modify a new disease, altering or modifying its appearance, course or sequelæ; this for a season or for a lifetime. In illustration of this, syphilis may be mentioned. It is, I belive, a view held by many that a successful inoculation having once taken place, a true indurated chancre having followed, the production of a similar sore under the influence of a new inoculation is almost impossible; even when it does appear it is modified, antagonized; it is only a "syphiloid" disease.

But perhaps, the most valuable illustrations of this law is in the action of one remedy in the system in modifying, or antagonizing, that of another. Our opulent materia medica is rich in illustrations of this law of drug antagonism. Thus strychnia is antagonized in its action on the system by chloral hydrate and the bromides; ether vapor by the action of cold; Calabar bean by that of atropia; belladonna by opium and jaborandi. And what are all these multiplied examples but illustrations of this great law of antagonism—the antagonism of drug action as well as of morbid action.

Again, how rich the field of illustration presented to us also in the study of *diathetic* influences over intercurrent diseases. Every day's clinical practice exemplifies this, and it furnishes us valuable practical points. The diathetic state is constantly stamping its modifying impress on intercurrent or acute specific diseases with which it may be associated, and we make little headway in the cure of the special disease until we correct the predisposing diathesis.

I can only suggest the thought; to follow it out would be to review the whole history of practical medicine; sometimes the influence may be one of cure, often however, like our drugs, it is the reverse.

But I aim only, in the limited space of my paper, briefly to show in these general statements the action and reaction of these morbid elements on each other, and to point out the fact that these actions may be in the direction of *antagonism*, and, therefore, curative; and by parity of reasoning, which is, I think, fair and logical in this instance, I arrive at the conclusion that drugs, when they act *curatively*, have the same modifying and counteracting influence, and that this *antagonism* is, in fact, their "*law of cure.*"

If we compare for a moment drug action with diseased action, we observe the same general law of physiological antagonism. Thus aconite, veratrum and quinia reduce fever; colchicum cures gout; mercury and iodine antagonize syphilis; quinia and arsenic arrest an intermittent; while strychnia, belladonna, digitalis and carbonate of ammonia are specific stimulants to the respiratory and cardiac nerve centers, thus rousing the heart and lungs into activity when embarassed from exhaustion, and enabling them to again respond to the natural stimuli to action.

How these agents accomplish these results we may not know, indeed we do not in the present state of medical knowledge. That their modes of action are many and various we have abundant proof, but one thing is sure, and that is, that a remedy, if introduced into the system in sufficient quantity to be perceived by it, never occupies neutral ground. It either coincides with or counteracts the morbid process, and that is what I mean by physiological antagonism; that is to say, that remedies, when they act *curatively* act *physiologically*—they so act on the ultimate dynamic life-forces of the system that morbid action is antagonized, and the system returns by its own intrinsic law of cure to its normal physiological type.

This view, it will be seen, recognizes at once the autocrasy of nature in the cure of disease; all remedies, to be curative, must act in harmony with nature; for if they do not they coincide with instead of antagonize the disease.

To this proposition, I think, no exception can be taken; it is universal in its application, and practical in its tendencies and results.

Thus we find rational medical science ever ready and willing to accept the actual results of careful and intelligent experimental observations. It must, for the present at least, remain the basis of all sound practical medicine. In other words, all sound practical experience, well attested and well established by the profession, must be accepted as *true science* whether we perceive the science or not.

It cannot, of course, be claimed that the materia medica of to-day is an exact science; then how necessary it is that the practitioner should be thoroughly grounded in some great fundamental underlying principles of this branch, that he may not merely treat the superficial symptoms or states of disease, but reach to the deeper causes and processes that produce them, and these he will find in the disturbed conditions of the processes by which we live, and our remedies can only act rationally and curatively when they counter-act or antagonize the disturbing agents. If it is possible, in the present state of medical science, to state any general law of cure, this *law of physiological antagonism* is perhaps as nearly correct as any formulated statement that can be made. This antagonism, it will be readily seen, may act in many and various ways; in some instances we may be able to explain its action, in others not; but in all instances we fall back on rational experience, which is at present the safest guide and surest rule of sound medical practice.

The future, we may reasonably hope, may reveal much more exactly the precise *how* and *why* of the action of remedies, their modes and processes in the system, their actions and counteractions; and it may be that we shall have much clearer conceptions as to the true scientific "law of cure" than we have at present; but I maintain that no matter how exact and accurate our knowledge may be, the law will be in all time that of antagonism; hence the formulated statement of my paper.

All remedies that act in harmony with nature in counteracting disease act, of course, on a principle *different* from the diseased action, and the law of cure, it logically follows, consists essentially in this *different action,* whatever it may be and however it may act.

On this general statement all "schools" can harmonize; all can stand on the common platform of rational empiricism until exact science can formulate a more definite and universal law of cure.

It may be objected to the views here presented that they are too general and do not tend to exact scientific observation of the action of remedies. In reply I have to say that I simply affirm the supremacy of *nature* in the cure of disease, and nature and science can never be in antagonism. A system of cure to be rational must be natural cure; it must act in harmony with nature. The aid we give nature may, it is true, sometimes consist in similar action, sometimes in contrary action; but it is safe to affirm that it must always be *different* action from that of the disease, and I therefore affirm that the real law of cure is that of Physiological Antagonism.

The cure takes place in obedience to this law of physiological antagonism, whether the active law in the individual case be that of "similar" action or "contrary" action. And the size of the dose must have reference to these two dissimilar actions. "If the relationship of the medicinal effects," says Kidd, "be analogous to the symptoms of the disease, the increased sensibility which this law of action begets calls for a *moderate* dose, *i. e*, less than the amount required to produce the full physiological effects. When the relationship is opposite or dissimilar to the symptoms of the diseases then full (large) doses are required, and more frequent repetition."

The process of cure is physiological; that is, remedies act by and through the same agency that effects spontaneous cures. The acts are such conservative operations as pertain to living organisms, and such a system I call, by way of distinction, "*natural cure*"

It is the healing art founded on natural principles, and these principles, acts or agencies, will be found, on careful analysis, to grow ont of a law of vital antagonism to the states or conditions which produce disease. The antagonism was recognized by the older writers as the "*vis medicatrix nature.*"—*Detroit Lancet.*

